# A View on the Synthesis and Characterization of Porous Microspheres Containing Pyrrolidone Units

**DOI:** 10.3390/ma18112432

**Published:** 2025-05-22

**Authors:** Małgorzata Maciejewska

**Affiliations:** Department of Polymer Chemistry, Institute of Chemical Sciences, Faculty of Chemistry, Maria Curie-Skłodowska University in Lublin, Gliniana 33, 20-614 Lublin, Poland; mmacieje@umcs.pl

**Keywords:** polymeric microspheres, porous structure, thermal behavior

## Abstract

Porous materials are used in many important applications, such as separation technologies, catalysis, and chromatography. They may be obtained from various monomers via diverse polymerization techniques and a wide range of synthesis parameters. The study is devoted to the synthesis and characterization of crosslinked porous polymeric spheres containing pyrrolidone subunits. To achieve this goal, two methods were applied: direct synthesis from *N*-vinyl-2-pyrrolidone (NVP) with ethylene glycol dimethacrylate (EGDMA) and via a modification reaction of porous poly(glycidyl methacrylate-*co*-ethylene glycol dimethacrylate) with pyrrolidone (P). The polymerization was carried out with the use of different molar ratios of the monomers. In order to obtain highly porous materials, pore-forming diluents (toluene, dodecane, and dodecan-1-ol) were used. The synthesized copolymers were characterized using size distribution analysis, ATR-FTIR spectroscopy, scanning electron microscopy, thermogravimetry, and inverse gas chromatography. Determined by the nitrogen adsorption/desorption method, the specific surface area was in the range of 55–468 m^2^/g. The good thermal properties of the poly(VP-*co*-EGDMA) copolymers allowed them to be applied as the stationary phase in gas chromatography.

## 1. Introduction

Porous microspheres are among the most advantageous materials. They find effective use in a multitude of applications, including column packing in various chromatography methods, filtration and separation membranes, support for chemical sensors and biosensors, catalysts, transporters in drug delivery systems, and tissue regeneration scaffolds [1,2,3,4,5,6,7,8,9]. They are also extensively employed in the detection and elimination of water contaminants, atmospheric carbon dioxide capture, hydrogen storage, separation of rare earth elements, atmospheric water harvesting, and several other advanced applications [10,11,12,13,14,15,16,17,18,19,20,21,22]. They are especially noteworthy when, alongside their porosity, they exhibit additional beneficial characteristics like thermal stability or chemical diversity. To achieve this goal, they are created from a variety of monomers using different polymerization methods and an extensive array of synthesis conditions. However, a significant category of porous polymers still remains lacking: materials obtained on the basis of non-polar monomers, e.g., poly(St-*co*-DVB) copolymer [23,24,25,26,27,28]. Despite its huge chemical stability, the copolymer’s surface exhibits hydrophobic characteristics, impeding wetting by aqueous solutions and restricting its interaction with polar molecules. Consequently, the adsorption of hydrophilic substances, including metal ions, during separation processes is strongly limited. Also, low efficiency in aqueous chromatography, e.g., in HPLC for polar analytes, is observed for the poly(St-*co*-DVB) stationary phase. What’s more, problems related to biocompatibility are evident, particularly in biotechnological applications where interaction with the aqueous environment is essential.

To enhance the effectiveness of porous microspheres, the incorporation of **functional monomers** that can introduce polarity and chemical reactivity into the polymer matrix has become an important approach. One such monomer is **N-vinyl-2-pyrrolidone (NVP)**. It offers both a hydrophilic character and biocompatibility. Its incorporation imparts **pyrrolidone groups** into the resulting copolymer network, which can improve specific interactions with polar molecules. This phenomenon noticeably increases their selectivity and affinity in applications like chromatography, specific sorption, and controlled release [29,30,31,32,33]. However, the synthesis of porous copolymers containing a high amount of NVP can be a challenge, particularly related to achieving a balance between **monomer incorporation and porous structure development.** Additionally, its hydrophilic character thereby constrains NVP use in aquatic environments. To overcome this problem, post-polymerization modification can be successfully applied. It offers an alternative and often more precise strategy to tailor the functionality of porous polymers. For instance, glycidyl methacrylate (GMA) is a reactive monomer whose epoxy groups can be chemically transformed post-polymerization [34,35,36]. By modifying GMA-based copolymers with pyrrolidone, it is possible to introduce pyrrolidone functionalities on the surface of the copolymer and maintain control over the porous structure [37,38]. This dual approach—direct copolymerization and chemical modification—enables a comparative assessment of material properties and the optimization of synthesis routes for targeted performance.

In this research, we present the synthesis, characterization, and thermal evaluation of novel porous materials bearing pyrrolidone units. Two complementary strategies were employed:

1. Direct suspension copolymerization of NVP with ethylene glycol dimethacrylate (EGDMA) under varying monomer ratios and diluent systems to explore the influence on morphology, porosity, and thermal stability.
2. Post-polymerization modification of poly(GMA-*co*-EGDMA) copolymers via reaction with pyrrolidone to evaluate the functionalization efficiency and preservation of the porous architecture.

The obtained materials were thoroughly characterized using scanning electron microscopy (SEM), ATR-FTIR spectroscopy, elemental analysis, nitrogen adsorption/desorption measurements (BET and BJH methods), thermogravimetric (TG/DTG) analysis, and gas chromatography (GC). Particular attention was given to how polymer composition and diluent polarity impact the nitrogen content, surface area, and thermal degradation behavior. Furthermore, the chromatographic performance of the synthesized materials was evaluated using mixtures of alcohols to validate their applicability in real-world separation systems.

This study aims to extend the understanding of how formulation and synthesis pathways influence the structure–property relationship of pyrrolidone-functionalized porous polymers and to identify material design principles for high-performance stationary phases in gas chromatography and related technologies.

## 2. Materials and Methods

### 2.1. Chemicals

In order to remove inhibitors, *N*-vinyl-2-pyrrolidone (NVP), 2,3-epoxypropyl methacrylate (GMA), and ethylene glycol dimethacrylate (EGDMA) (Sigma-Aldrich, Steinheim, Germany) were cleaned using 4% aqueous sodium hydroxide. 2,2′-azoisobutyronitrile (AIBN) and poly(vinyl alcohol) (PVA) (both purchased from Fluka AG, Buchs, Switzerland) were applied without any purification. Reagent grade dodecane, decan-1-ol, toluene, acetone, and methanol were purchased from POCh (Gliwice, Poland). Pyrrolidone (P) was bought from Sigma-Aldrich (Steinheim, Germany).

### 2.2. Syntheses of the Porous Microspheres

The aqueous suspension was applied as the polymerization medium. During the course of copolymerization, distilled water (195 mL) and PVA (6.5 g) were mixed for 5 h at a temperature of 80 °C in a three-necked flask equipped with a thermometer and water condenser. After the dissolution of PVA (a suspension stabilizer), the organic mixture of 15 g of monomers and 0.15 g of azobisisobutyronitrile (AIBN, 1.0 wt% relative to the total monomer mass), as the radical initiator, in 22.5 mL of proper diluent was incorporated into the water phase. The weight-to-volume ratio between the monomers and the diluents was maintained at 1:2 *w*/*v*. The obtained suspension was vigorously mixed at 300 rpm and maintained at 80 °C for 20 h under an air atmosphere. The porous microspheres produced during this process were filtered and subjected to an extensive cleaning procedure described earlier [39]. The cleansed microspheres were dried overnight under vacuum at 80 °C. Table 1 presents the experimental parameters and the designations of the resulting copolymers.

### 2.3. Measurement Techniques

The characterization of the synthesized copolymers was conducted using FTIR-ATR spectroscopy. The spectra were collected using a Bruker Tensor 27 FTIR spectrometer (Ettlingen, Germany). The spectral range was equal to 4000–600 cm^−1^. Each spectrum was acquired with 16 scans at a resolution of 4 cm^−1^. FTIR -ATR measurements were recorded using a diamond crystal.

Elemental analysis of the synthesized copolymers was conducted using a Perkin-Elmer (Norwalk, CT, USA) CHN analyzer.

The characterization of the porous structure of the analyzed materials was established on the basis of nitrogen adsorption/desorption measurements. They were made volumetrically with the use of an ASAP 2405N analyzer (Micromeritics Corp., Norcross, GA, USA). Before measurement, the samples were outgassed at 110 °C for 2 h. The nitrogen isotherms were collected at 77 K. The specific surface area (*S_BET_*), total pore volume (*V*), and pore size distribution (PSD) were determined on the basis of BET and BJH methods [40].

The SEM imaging of the copolymers was conducted utilizing a Duall BeamTM, Quanta3D FEG microscope (FEI Company, Hillsboro, OR, USA). The size distribution of the copolymers was assessed using a Mastersizer Analyzer 2000 (Malvern Instruments Ltd., Malvern, UK).

A Netzsch STA 449 F1 Jupiter thermal analyzer was utilized for the thermogravimetric (TG) measurements. The analyses were conducted in the temperature range 35–800 °C utilizing Al_2_O_3_ crucibles. An empty Al_2_O_3_ crucible, with a mass of approximately 160 mg, was utilized as a reference. The mass of the samples was approximately 10 mg. The TG curves were analyzed to determine the characteristic temperatures of T_5%_, T_20%_, and T_50%_ mass losses. Consequently, on the basis of differential curves (DTG), the temperature corresponding to the maximum mass loss (T_max_) for each decomposition stage was assessed.

A Dani GC 1000 gas chromatograph (Dani, Milan, Italy) was employed for the chromatographic measurements. It was equipped with a packed injector set at 210 °C, and a thermal conductivity detector (TCD) also maintained at 210 °C. The flow of the carrier gas (helium) was maintained at 50 mL/min. The samples were injected by hand with a 1 μL syringe (SGE, North Melbourne, Australia). Measurements were performed at 140 °C and 200 °C.

## 3. Results and Discussion

**Figure 1:**
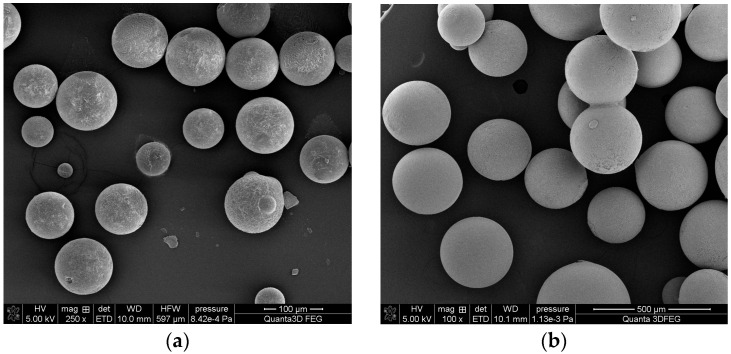
SEM photographs of the investigated microspheres: (**a**) poly(GMA-*co*-EGDMA)_1 copolymer; (**b**) poly(NVP-*co*-EGDMA)_1 copolymer.

Crosslinked porous polymers can be synthesized via a range of polymerization techniques. In this study, a suspension polymerization technique was chosen because it enabled the synthesis of porous microspheres with diameters of approximately 100 µm (Figure 1). Such a diameter is required in many essential techniques like PALS studies, gas chromatography, catalysis, etc. However, a serious drawback of this technique is the quite broad size distribution of the synthesized microspheres [41].

**Figure 2:**
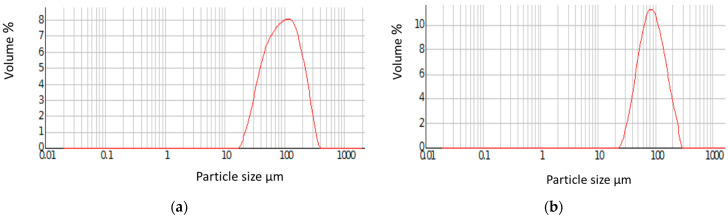
Relative distributions of the microsphere volume as a function of diameter: (**a**) poly(GMA-*co*-EGDMA)_1 copolymer; (**b**) poly(NVP-*co*-EGDMA)_1 copolymer.

Figure 2 presents two graphs that compare the relative distributions of microsphere volume as a function of diameter for the poly(GMA-*co*-EGDMA)_1 and poly(NVP-*co*-EGDMA)_1 copolymers. The graphs depict the particle size distributions, showing how the volume percentage is distributed over different particle sizes. The overall shape of both distributions exhibited a log-normal pattern, as expected in suspension polymerization. The tailing effect was minimal. The maximum of the peak, which represented the most frequent particle size in the distribution of poly(GMA-*co*-EGDMA)_1, was visible at about 100 µm. For the poly(NVP-*co*-EGDMA)_1 copolymer, the peak was slightly shifted and occurred at about 80 µm. It was well defined with less tailing. What’s more, the particle size distribution was narrower, meaning that most particles fell within a relatively confined size range. Generally, better uniformity in microsphere size is more desirable in most applications. As is shown, the application of the NVP functional monomer in polymer synthesis provided better size control compared with the use of GMA.

**Figure 3:**
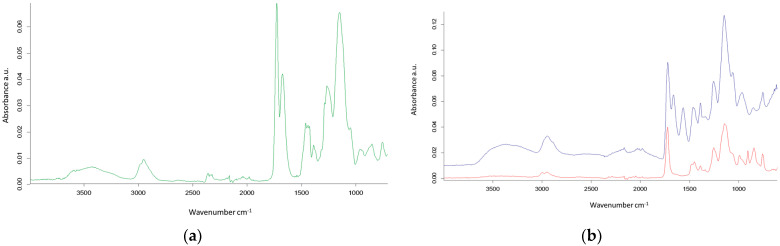
ATR spectra of the investigated copolymers: (**a**) poly(VP-*co*-EGDMA)_1 (green line); (**b**) parent poly(GMA-*co*-EGDMA)_1 (red line), modified poly(GMA-*co*-EGDMA)_1+p (blue line).

The main objective of the presented study was to synthesize porous microspheres containing pyrrolidone units. In the first part of the study, copolymerization of N-vinyl-2-pyrrolidone with ethylene glycol dimethacrylate was performed. The molar ratio of NVP to EGDMA used in the synthesis was progressively adjusted from an initial 1:1 up to 4:1. The proper course of polymerization was monitored with the use of FTIR-ATR spectroscopy (Figure 3).

**Figure 4:**
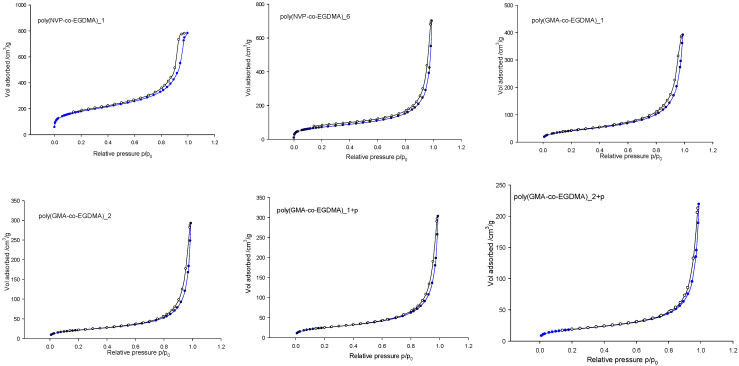
The isotherms of nitrogen adsorption (●) and desorption (○) on selected copolymers determined at 77 K.

In the first panel (a) of Figure 3, the ATR spectrum of poly(VP-*co*-EGDMA)_1 is presented. As can be seen, the bands characteristic of alkyl and alkylene groups, i.e., at 2962–2951 cm^−1^ (ν C–H) and 1459–1425 cm^−1^ (δ C–H), are present. The occurrence of the ester group is revealed by the band at 1726 cm^−1^ (ν C=O) and confirmed by the bands at 1196–1087 cm^−1^ (ν C–O). The band at 1673 cm^−1^ is related to the γ-lactam ring present in NVP (the C=O stretching vibrations). Additionally, the presence of pyrrolidone units was confirmed by elemental analysis. The synthesized copolymers contained from 2.8 to 6.4% nitrogen depending on the molar ratio and diluent type (Table 2). As expected, the increment in NVP quantity in the polymerization mixture led to an elevated nitrogen proportion in the synthesized copolymer. The determined nitrogen percentage for poly(NVP-*co*-EGDMA)_1 copolymer was 3.01, whereas for poly(NVP-*co*-EGDMA)_8, in which the molar ratio of NVP to EGDMA equaled 4:1, it increased to 6.40. The presented values were lower than theoretically calculated values and indicated that only about 70% of the NVP initial amount was incorporated into the polymeric matric. However, this value was much higher in comparison to the poly(NVP-*co*-DVB) copolymer investigated in an earlier study [40]. The results for the copolymers derived from equimolar ratios of monomers revealed the impact of diluent composition. The lowest nitrogen amount was found in the copolymer synthesized in the presence of toluene/dodecane mixture (2.80%). The application of a more polar diluent (decan-1-ol) instead of dodecane resulted in an increase in the amount of nitrogen and consequently in the pyrrolidone unit content in the poly(NVP-*co*-EGDMA)_2 and poly(NVP-*co*-EGDMA)_3 materials. The diluent composition also had a great impact on porous structure formation. Nitrogen sorption analysis was employed to determine the key parameters of the porous structure for all copolymers studied. The isotherms of nitrogen adsorption/desorption on selected copolymers are presented in Figure 4.

The copolymer with the most developed specific surface (456 m^2^/g) area was obtained when toluene was applied as the pore-forming diluent (Table 2).

This behavior could be clarified on the basis of the toluene solubility parameter, which was equal to 18.2 (MPa)^0.5^. This value indicated the good solvation power of toluene. In an initially homogenous polymerization system containing a good solvent, phase separation (indispensable for the formation of porous morphology) occurred at a greater conversion of monomers to polymer. During this late phase separation, a huge number of individual nuclei were created. Toluene competed with the monomer for solvation within the nuclei, resulting in a relatively low local concentration of monomers inside the nuclei. Attractive forces between individual nuclei were weak, thus limiting coalescence. Consequently, small globules were formed. They were interconnected and created a polymeric network with a high surface area. The pore size distribution had the maximum in the region of micro- to mesopores. The incorporation of a poorer solvent (dodecane) led to earlier phase separation because of the limited solubility of the polymer phase in the polymerization mixture. The newly created phase favorably swelled with the monomers. The monomer concentration within the nuclei was relatively high. It led to coalescence and a subsequent increase in their size. As a result, the formed globules and the free voids between them were larger. Consequently, larger pores and a less developed specific surface area were noticed for the poly(NVP-*co*-EGDMA)_4 copolymer. A further increment in the dodecane concentration in the organic mixture led to further diminution of the specific surface area, as can be seen for the poly(NVP-*co*-EGDMA)_5 copolymer. Evidently, the solvent controlled copolymer porosity via polymer chain solvation in the early stages of polymerization. The porosity of the synthesized copolymers was also strongly dependent on the molar ratio of the comonomers. In systems with elevated crosslinker content, the onset of phase separation was accelerated. However, the higher crosslinker density of the nuclei limited their ability to adsorb additional monomer. In addition, increased crosslinker density suppressed the tendency of particles to coalesce. As a result, the large number of small nuclei remained large, and it was responsible for the highly developed internal structure. A reduction in the crosslinker content within the polymerization medium led to a decrease in the specific surface area. The copolymers synthesized with a higher amount of functional monomer were characterized by a larger content of nitrogen in their structure but a considerably lower porous structure. This parameter decreased from 456 m^2^/g for poly(NVP-*co*-EGDMA)_1 to 56 m^2^/g for poly(NVP-*co*-EGDMA)_8. Relatively polar monomers bearing both hydrophilic and hydrophobic units (e.g., NVP) increased the agglomeration tendency of crosslinked nuclei. The nuclei grew together. The loss of structural distinction among the particles corresponded with lower porosity. A similar situation was observed in the case of the application of more polar diluent (decan-1-ol). Compared with apolar dodecane, it promoted the incorporation of NVP into the polymeric matrix but led to a diminution of the specific surface area to about 80 m^2^/g.

Another approach to obtain polymers with a developed internal structure and bearing pyrrolidone units is the production of poly(GMA-*co*-EGDMA) copolymers and their modification with pyrrolidone. The processes of polymerization and subsequent modification were monitored using infrared spectroscopy. In the second panel (b) of Figure 3, the spectra of the parent poly(GMA-*co*-EGDMA)_1 (red line) and modified pyrrolidone poly(GMA-*co*-EGDMA)_1+p (blue line) copolymers are displayed. In the ATR spectrum of the functionalized copolymer, significant reductions in the peak absorbances at 906 and 844 cm^−1^, related to the epoxy ring, are visible. Meanwhile, a new peak at 1663 cm^−1^ emerges, attributed to pyrrolidone subunits, indicating the successful transformation of epoxy groups into pyrrolidone. Furthermore, a broad peak within the range of 3290–3300 cm^−1^ related to O–H stretching vibrations can be seen.

Additionally, the modification step influenced the porosity of the resulting copolymers. The porous structure of the modified copolymers slightly decreased. This observation was in accordance with previous ones.

**Figure 5:**
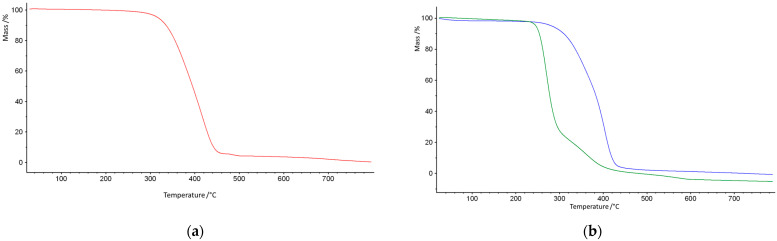
TG curves of the investigated copolymers: (**a**) poly(NVP-*co*-EGDMA)_1 (red line); (**b**) parent poly(GMA-*co*-EGDMA)_1 (green line) and modified poly(GMA-*co*-EGDMA)_1+p (blue line).

**Figure 6:**
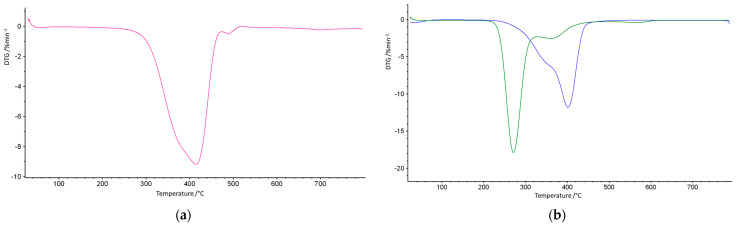
DTG curves of the investigated copolymers: (**a**) poly(NVP-*co*-EGDMA)_1 (pink line); (**b**) parent poly(GMA-*co*-EGDMA)_1 (green line), modified poly(GMA-*co*-EGDMA)_1+p (blue line).

As some of the potential applications of porous microspheres require good thermal stability, it was of interest to investigate their thermal behavior. It was evaluated on the grounds of TG and DSC methods. Figure 5 displays the TG curves of the investigated copolymers, whereas their first derivatives are presented in Figure 6.

On the basis of their characteristic temperatures, the T_5%_, T_20%_, and T_50%_ mass losses as well as the maxima of mass loss were determined and are collected in Table 3.

**Figure 7:**
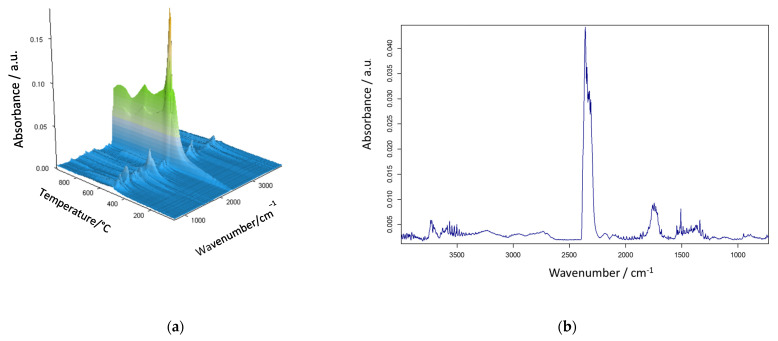
Infrared spectra of gaseous products released during thermal degradation of poly(VP-*co*-EGDMA)_1: (**a**) recorded across the full heating range and (**b**) extracted at the maximum of degradation (417 °C).

As follows from the presented results, the best thermal resistance was obtained for the poly(NVP-*co*-EGDMA)_1 copolymer. Its decomposition began at a temperature exceeding 300 °C, and the temperatures of 20% and 50% mass loss were the highest among all of the studied copolymers. What is important is that the decomposition process proceeded in a single stage. The poly(GMA-*co*-EGDMA)_1 copolymer demonstrated a reduced thermal resistance. It started to decompose at about 250 °C, and this process underwent two steps, with maxima at 272 and 369 °C. Although the main thermal degradation step corresponded to the first maximum. Increasing the amount of functional monomer into the polymeric matrix (poly(GMA-*co*-EGDMA)_2 copolymer) resulted in a further decrease in its thermal properties. The opposite effect was triggered by the modification process. The modification process with pyrrolidone improved the thermal resistance of the materials under study. The T_5%_ parameter increased by about 30 °C for both modified copolymers. The changes were even more profound with respect to the maxima of decomposition. After modification, T_1max_ increased by 78 °C for the poly(GMA-*co*-EGDMA)_1+p copolymer and by 69 °C for poly(GMA-*co*-EGDMA)_2+p. For these copolymers, the major mass loss occurred during the second decomposition stage. The decomposition of the copolymers was monitored by infrared spectroscopy to identify the evolved gases. Figure 7 presents the FTIR spectra of the gaseous degradation products emitted during the heating of poly(VP-*co*-EGDMA)_1. **In this copolymer, NVP-based units** promoted α-scission due to **polar functional groups** that **stabilized transition states** or favored radical delocalization. The main products of α-hydrogen bond scission are carbon oxide, carbon dioxide, water, and aldehydes. Aldehydes are formed when α-scission occurs near the ester group between the backbone and the ester carbon.

Consequently, in Figure 7, the bands characteristic of the asymmetric stretching vibrations of CO_2_ (at 2359–2310 cm^−1^), CO (at 2181 and 2114 cm^−1^), and H_2_O (at ~4000–3500 cm^−1^), as well as the bending vibrations of H_2_O (at ~1800–1300 cm^−1^), are noticeable. Additionally, carbonyl absorption in the range of 1772–1770 cm^−1^, corresponding to C=O stretching, is evident and is likely due to α-hydrogen bond cleavage.

**Figure 8:**
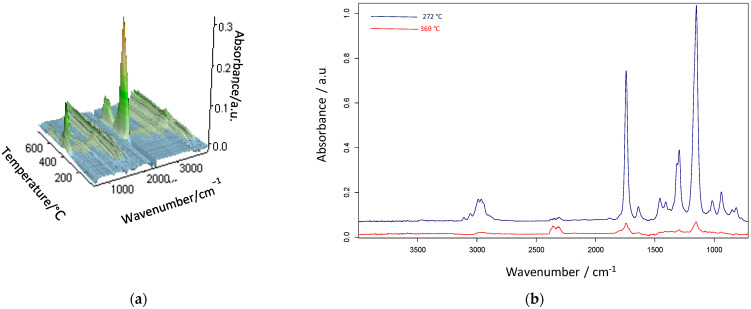
Infrared spectra of gaseous products released during thermal degradation of poly(GMA-*co*-EGDMA)_ 1: (**a**) recorded across the full heating range and (**b**) extracted at the temperatures of degradation maxima.

In Figure 8, the infrared spectra of the gaseous products released during the thermal degradation of poly(GMA-*co*-EGDMA)_1 recorded across the full heating range (a) and extracted at temperatures of the degradation maxima (b) are presented. As can be seen, the spectrum obtained at the first degradation maximum exhibited absorption bands characteristic of carbonyl compounds, including -C=O stretching at 1742 cm^−1^ and -C–O stretching at 1150 cm^−1^. Evidence of vinyl compound formation was also observed in the spectrum, with absorption bands at 3110 cm^−1^ (-C–H stretch), 939 and 1017 cm^−1^ (-C–H out-of-plane deformation), and 1693 cm^−1^ (-C=C stretch). The presence of aliphatic groups was supported by spectral bands at 2964 and 2989 cm^−1^. They were related to the symmetric and asymmetric C–H stretching vibrations of the methylene and methyl groups. What is extremely important is that an oxirane-related peak at 850 cm^−1^ is clearly visible. This suggests that during the first degradation step, depolymerization occurs and emission of the GMA monomer takes place. The spectrum of the parent copolymer collected at 272 °C is analogous with the GMA spectrum in the NIST database [42]. In the spectrum recorded at the second maximum of degradation, the bands connected with CO_2_ stretching vibrations (2360–2311 cm^−1^) and CO stretching vibrations (2181 and 2114 cm^−1^) are mainly visible. Additionally, ester-related vibrations at 1745 cm^−1^ (-C=O stretching) and at 1154 cm^−1^ (-C–O stretching) are present. The absorption bands mentioned above suggested that the thermal degradation of the poly(GMA-*co*-EGDMA) copolymers was driven by the breakdown of their ester functionalities. The infrared spectra of gases that evolved during the heating of the modified poly(GMA-*co*-EGDMA)_1+P copolymer are presented in Figure 8. The spectrum collected at the first maximum of decomposition (350 °C) shows bands associated with CO_2_ (2359–2310 cm^−1^) and CO (2186 cm^−1^) as well as carbonyl-related (1748 cm^−1^ and 1133 cm^−1^) and water-related (4000–3500 cm^−1^ and 1700–1300 cm^−1^) bands. What’s more, bands that can be connected with the appearance of amide (3587–3567 cm^−1^ and 1767 cm^−1^) are noticeable. In the second spectrum, extracted at the maximum of degradation (417 °C), mainly water and carbon dioxide are visible.

**Figure 9:**
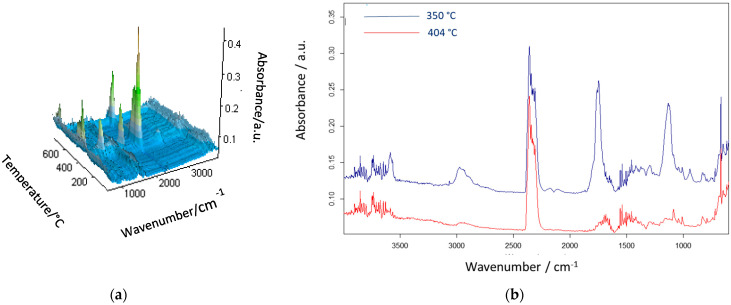
Infrared spectra of gaseous products released during thermal degradation of poly(GMA-*co*-EGDMA)_1+p: (**a**) recorded across the full heating range and (**b**) extracted at the temperatures of degradation maxima.

Figure 9 shows infrared spectra of gaseous products released during thermal degradation of poly(GMA-*co*-EGDMA)_1+p copolymer. The characteristic absorption bands observed in FTIR spectra of the all investigated copolymers are summarized in Table S1.

The thermal decomposition behavior of the investigated copolymer elucidated using TG-FTIR analysis of the evolved gases confirmed a multi-step degradation mechanism under inert (helium) conditions, consistent with the behavior of other functional methacrylate-based microspheres. The infrared spectra of gases collected throughout the heating program revealed distinct absorption bands corresponding to carbon monoxide, carbon dioxide, and water vapor, along with organic fragments showing C=O, N–H, and vinyl (C=C) vibrations, suggesting progressive breakdown of the pyrrolidone ring and the main backbone.

In light of recent findings [43] on thiol-functionalized GMA copolymers, a self-decomposition mechanism appears plausible in our nitrogen-containing systems as well. In the referenced study, decomposition was initiated by transesterification between thiol and ester groups, producing carbonyl sulfide (COS), acrolein, and CO_2_, with subsequent fragmentation driven by radical pathways. Analogously, in our system, the presence of –C=O and –N–H bonds in pyrrolidone may promote intramolecular hydrogen abstraction and radical-driven cleavage, initiating degradation without the need for external oxidants. These polar groups likely lower the activation energy for chain scission, enabling decomposition to begin at relatively modest temperatures.

Notably, the FTIR spectra obtained during decomposition revealed strong bands associated with amide carbonyls (around 1680–1720 cm^−1^) and decomposition products such as acrolein (2810–2780 cm^−1^), indicating that ring-opening and chain unzipping dominate the early stages of decomposition. As in thiol-containing systems, we propose that decomposition proceeds via (i) endothermic scission of methacrylate backbones, (ii) partial oxidation and isomerization of the pyrrolidone ring, and (iii) release of small-molecule volatiles (CO, CO_2_, and aldehydes), culminating in a carbon-rich, unsaturated residue.

These findings suggest that pyrrolidone-based copolymers undergo a partially self-initiated, multi-step decomposition mechanism with a significant contribution from polar, nitrogen-containing functionalities, thereby corroborating the self-decomposition hypothesis under non-oxidative conditions.

**Figure 10:**
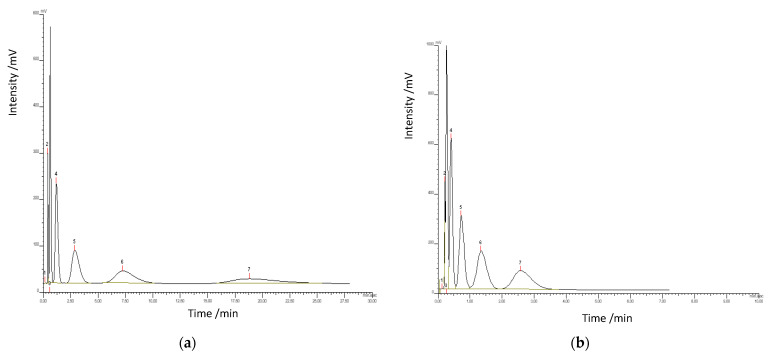
GC chromatograms of alcohol separation performed at (**a**) 140 °C and (**b**) 200 °C using the poly(NVP-*co*-EGDMA) copolymer as the stationary phase.

The good thermal stability exhibited by the synthesized copolymers offers an opportunity for their application in high temperature techniques, e.g., gas chromatography. Furthermore, the incorporation of pyrrolidone subunits in the copolymer matrix imparts hydrophilic characteristics. The microspheres offer balanced polarity—enough to interact with analytes, but not so polar as to cause strong, irreversible adsorption. They enable specific polar interactions via hydrogen bonding (N–H and C=O), dipole–dipole interactions, and Lewis acid–base interactions with polar analytes. These molecular recognition features are essential for separating chemically similar organic volatile compounds—e.g., isomers, alcohols, and polar ketones—where subtle differences in polarity and hydrogen bonding capabilities are crucial. This results in higher resolution, better peak separation, and enhanced selectivity. Figure 10 presents the chromatograph of a mixture of aliphatic alcohols analyzed at temperatures of 140 °C and 200 °C. It is evident that, at elevated temperatures, the duration needed for alcohol separation was significantly reduced, and the peaks exhibited less diffusion. Consequently, thermally stable copolymers offer greater benefits for GC techniques.

## 4. Conclusions

In this research, a series of porous copolymers bearing pyrrolidone units was successfully synthesized using the suspension polymerization technique. The investigation focused on how variations in monomer ratio, diluent type, and post-synthetic modification influenced the thermal and physicochemical properties of the resulting microspheres. Among the synthesized materials, poly(NVP-*co*-EGDMA)_1 demonstrated superior porous characteristics, exhibiting the highest specific surface area (456 m^2^/g) and excellent thermal stability, with decomposition onset above 300 °C.

Increasing the NVP amount in the polymerization mixture led to enhanced incorporation of pyrrolidone units, although at the cost of reduced porosity. The type of diluent employed during polymerization also played a pivotal role: polar solvents like decan-1-ol favored NVP incorporation but diminished textural parameters, while toluene promoted high surface area formation due to delayed phase separation.

Moreover, an alternative route—post-polymerization modification of poly(GMA-*co*-EGDMA) copolymers with pyrrolidone—allowed for the incorporation of pyrrolidone functionalities while preserving a considerable degree of porosity. This modification not only improved pyrrolidone incorporation but also notably enhanced thermal resistance.

Thermal analysis revealed that copolymers with NVP exhibited single-stage decomposition, a trait favorable for thermal applications, whereas GMA-based materials underwent multi-stage degradation, partially due to monomer release during depolymerization. FTIR studies of the evolved gases confirmed the emission of typical decomposition products such as CO_2_, CO, and aldehydes.

Finally, gas chromatography tests confirmed that pyrrolidone-bearing copolymers provided effective separation of polar analytes like alcohols, especially at elevated temperatures. The synthesized microspheres thus present great potential for chromatographic applications and other domains requiring thermally stable, polar-functionalized porous polymers.

## Figures and Tables

**Table 1:** Specifications and experimental conditions for the microsphere synthesis.

Copolymer	Molar Ratio of Monomers			Diluents/mL		
	EGDMA	VP	GMA	Toluene	Decan-1-ol	Dodecane
poly(NVP-*co*-EGDMA)_1	1	1	-	22.5	-	-
poly(NVP-*co*-EGDMA)_2	1	1	-	19.1	3.4	-
poly(NVP-*co*-EGDMA)_3	1	1	-	11.25	11.25	-
poly(NVP-*co*-EGDMA)_4	1	1	-	19.1	-	3.4
poly(NVP-*co*-EGDMA)_5	1	1	-	11.25	-	11.25
poly(NVP-*co*-EGDMA)_6	1	2	-	22.5	-	
poly(NVP-*co*-EGDMA)_7	1	3	-	22.5	-	-
poly(NVP-*co*-EGDMA)_8	1	4	-	22.5	-	-
poly(GMA-*co*-EGDMA)_1	1	-	1	22.5	-	-
poly(GMA-*co*-EGDMA)_2	2	-	1	22.5	-	-

**Table 2:** The basic parameters of porous structure and nitrogen weight fraction of the investigated copolymers.

Copolymer	Specific Surface Area *S_BET_* (m^2^/g)	Pore Volume *V* (cm^3^/g)	Pore Diameter *D_BJH_* (nm)	N %
poly(NVP-*co*-EGDMA)_1	456	0.675	31	3.01
poly(NVP-*co*-EGDMA)_2	88	0.624	31	3.45
poly(NVP-*co*-EGDMA)_3	76	0.313	50	3.60
poly(NVP-*co*-EGDMA)_4	267	0.566	37	2.80
poly(NVP-*co*-EGDMA)_5	190	0.275	40	2.97
poly(NVP-*co*-EGDMA)_6	101	0.215	37	3.80
poly(NVP-*co*-EGDMA)_7	81	0.284	38	4.40
poly(NVP-*co*-EGDMA)_8	59	0.319	47	6.40
poly(GMA-*co*-EGDMA)_1	109	0.483	39	-
poly(GMA-*co*-EGDMA)_2	72	0.280	52	-
poly(GMA-*co*-EGDMA)_1+p	101	0.378	38	1.51
poly(GMA-*co*-EGDMA)_2+p	68	0.210	51	2.50

**Table 3:** TG and DTG data of the copolymers determined in a helium atmosphere.

Copolymer	T_5%_ (°C)	T_20%_ (°C)	T_50%_ (°C)	T_1max_ (°C)	T_2max_ (°C)
poly(NVP-*co*-EGDMA)_1	317	355	394	417	-
poly(GMA-*co*-EGDMA)_1	251	265	288	272	369
poly(GMA-*co*-EGDMA)_2	245	257	276	265	367
poly(GMA-*co*-EGDMA)_1+p	283	337	385	350	404
poly(GMA-*co*-EGDMA)_2+p	277	301	348	334	370

## Data Availability

The data presented in this study are available upon request from the corresponding author.

## Supplementary Materials

The following supporting information can be downloaded at: https://www.mdpi.com/article/10.3390/ma18112432/s1, Table S1: FTIR Vibrational Mode Assignments.
