# Supplementary material for: A View on the Synthesis and Characterization of Porous Microspheres Containing Pyrrolidone Units
Source: Materials (Basel). 2025 May 22;18(11):2432. doi: 10.3390/ma18112432 (PMC12156811; doi:10.3390/ma18112432)
Supplement: Supplementary file 1 [file materials-18-02432-s001.zip › materials-3585884-supplementary.pdf]

Table S1. FTIR Vibrational Mode Assignments

Wavenumber (cm^{-1})	Vibrational Mode Assignment
669 cm^{-1}	δ C–O, bending in CO_2
760-750 cm^{-1}	δ C–O in epoxide ring
880-840 cm^{-1}	ω C–H waging in epoxide ring
950-915 cm^{-1}	ν C–O stretching in epoxide ring
920-900 cm^{-1}	δ =C–H bending (vinyl)
1005-958 cm^{-1}	γ =C-H out-of-plane
1196–1087 cm^{-1}	ν C–O, stretching in esters
1370–1380 cm^{-1}	δ_s CH_2 scissoring (in-plane bending)
1459-1425	δ C–H, bending in alkyl and alkylene group
1663 cm^{-1}	ν C=O stretching (pyrrolidone after modification)
1693 cm^{-1}	ν C=C stretching (vinyl groups)
1750–1735 cm^{-1}	ν C=O stretching (ester)
1767 cm^{-1}	ν C=O stretching (amide)
2186-2114 cm^{-1}	ν C–O asymetric stretching in CO
2359–2310 cm^{-1}	ν C–O, asymmetric stretching in CO_2
2820 & ~2720	ν C–H doublet in aldehyde
2975-2950 cm^{-1}	ν C–H asymmetric streching in alkyl and alkylene group
2885-2860 cm^{-1}	ν C–H symmetric streching in alkyl group
2935-2915	ν C–H asymmetric streching in alkylene group
2865-2840	ν C–H symmetric streching in alkylene group
3110 cm^{-1}	ν =C–H stretching (vinyl)
3587–3567 cm^{-1}	ν N–H stretching (amide)
